# Supplementary material for: Diagnostic value of metagenomic next-generation sequencing using bronchoalveolar lavage fluid samples for pathogen detection in children with severe or refractory pneumonia
Source: Microbiol Spectr. 2025 Feb 4;13(3):e01087-24. doi: 10.1128/spectrum.01087-24 (PMC11878053; doi:10.1128/spectrum.01087-24)
Supplement: Table S1 [file spectrum.01087-24-s0001.pdf]

Table S1： Original data of 127 pediatric patients

Patient	Underlying diseases	Age (y=years, m=months)	Gender（M=Male,F=Female）	Culture	mPCR	MP-IgM	MP IgG antibody titer	T-spot	Acid-fast staining (AFS)	Galactomannan (GM) test	Pathogens detected by DNA mNGS (relative abundance,%)	Pathogens detected by RNA mNGS(relative abundance,%)	Clinical diagnostic pathogens（Pathogenic considerations）	RMPP	MP resistance gene mutations detected by DNA mNGS	Changed antibiotics (refered results) , YES=effective, NO=ineffective）
1	No	4y	M	Negative	NA	Negative	NA	NA	Negative	NA	Mycoplasma pneumoniae-68(12.93)|Human bocavirus type I-1052(99.91)	Human bocavirus type I-5(35.71)	Mycoplasma pneumoniae (Azithromycin treatment was effective), Human bocavirus	NO	NO	Unchanged
2	No	7y	F	Negative	NA	Positive	＞1：1280	Negative	Negative	NA	Mycoplasma pneumoniae-604150(99.99)	Mycoplasma pneumoniae-35515(99.59)	Mycoplasma pneumoniae	NO	A2063G	Unchanged
3	No	3y	M	Negative	NA	Positive	1：1280	Negative	Negative	NA	|Mycoplasma pneumoniae-12585(89.16)|Candida tropicalis-10(5.29)	Mycoplasma pneumoniae-7957(98.08)	Mycoplasma pneumoniae	NO	NO	Unchanged
4	No	7y	F	Negative	NA	Positive	1:640	NA	Negative	NA	Haemophilus parainfluenzae-18(0.011)|Staphylococcus aureus-7(0.0044)Streptococcus pneumoniae-231(0.14)|Tropheryma whipplei-76(0.048)|Mycoplasma pneumoniae-39530(24.81)|Human betaherpesvirus 7-5(100.00)	Mycoplasma pneumoniae-70640(96.97)|RhinovirusA-3(13.63)	Mycoplasma pneumoniae	NO	A2063G	Unchanged
5	No	3y	F	Negative	NA	Positive	1：1280	Negative	Negative	NA	Mycoplasma pneumoniae-34832(98.69)	Mycoplasma pneumoniae-7769(97.31)	Mycoplasma pneumoniae	NO	A2063G	Unchanged

6	No	2y	F	Negative	NA	Positive	1：1280	NA	Negative	NA	Staphylococcus aureus-2(0.025)|Mycoplasma pneumoniae-5336(66.88)|Candida parapsilosis-2(2.86)	Mycoplasma pneumoniae-17328(98.55)	Mycoplasma pneumoniae	NO	NO	Unchanged
7	No	6y	F	Negative	Mycoplasma pneumoniae	Positive	>1：1280	Negative	Negative	NA	Mycoplasma pneumoniae-26291(99.84)|Human gammaherpesvirus 4-4(100.00)	Mycoplasma pneumoniae-623(77.78)|Human gammaherpesvirus 4-65(92.86)|RhinovirusB-3(4.29)	Mycoplasma pneumoniae, Rhinovirus-B	NO	A2063G	Unchanged
8	No	6y	F	Negative	Mycoplasma pneumoniae	Positive	>1：1280	Negative	Negative	NA	Mycoplasma pneumoniae-519433(99.99)|Corynebacterium resistens-5(0.001)	Mycoplasma pneumoniae-5302(86.75)	Mycoplasma pneumoniae	YES	A2063G	Unchanged
9	No	6y	F	Negative	Mycoplasma pneumoniae	Positive	>1：1280	Negative	Negative	NA	Mycoplasma pneumoniae-14250(99.89)	Mycoplasma pneumoniae-6286(91.25)	Mycoplasma pneumoniae	NO	NO	Unchanged
10	No	7y	M	Negative	Mycoplasma pneumoniae	Negative	Negative	Negative	Negative	NA	Haemophilus influenzae-11(7.97)|Mycoplasma pneumoniae-21(15.22)|Acinetobacter pittii -4(2.90)	Negative	Mycoplasma pneumoniae	NO	NO	Unchanged
11	No	7y	F	Negative	Mycoplasma pneumoniae	Positive	>1：1280	Negative	Negative	NA	Haemophilus parainfluenzae-6(3.85)|Mycoplasma pneumoniae-105(67.31)	Mycoplasma pneumoniae-6(0.93)	Mycoplasma pneumoniae	NO	NO	Unchanged
12	No	6y	M	Negative	Mycoplasma pneumoniae	Positive	>1：1280	Negative	Negative	NA	Haemophilus parainfluenzae-43(0.02)|Tropheryma whipplei-17(0.0085)|Mycoplasma pneumoniae-198750(99.74)	Mycoplasma pneumoniae-24974(96.02)	Mycoplasma pneumoniae	NO	A2063G	Unchanged

13	No	7y	F	Negative	Mycoplasma pneumoniae	Positive	>1：1280	Negative	Negative	NA	Mycoplasma pneumoniae-8602(99.99)	Mycoplasma pneumoniae-31489(98.05)	Mycoplasma pneumoniae	NO	NO	Unchanged
14	No	11m	F	Negative	Negative	Negative	Negative	Negative	Negative	NA	Mycoplasma pneumoniae-20(7.17)|Streptococcus pseudopneumoniae-3(1.08)	Negative	Mycoplasma pneumoniae (Azithromycin treatment was effective)	NO	NO	Unchanged
15	No	7y	M	Negative	NA	Positive	Negative	NA	Negative	NA	Mycoplasma pneumoniae-24426(99.89)	Mycoplasma pneumoniae-19437(99.15)|Human respirovirus 3-21(100.00)	Mycoplasma pneumoniae, Human parainfluenza virus	NO	A2063G	Unchanged
16	No	9y	F	Negative	NA	Negative	1：1280	NA	Negative	NA	Negative	Negative	Mycoplasma pneumoniae	NO	NO	Unchanged
17	No	3y	F	Negative	NA	Negative	>1：1280	Negative	Negative	NA	Mycoplasma pneumoniae-9410(99.80)	Mycoplasma pneumoniae-8233(98.67)	Mycoplasma pneumoniae	NO	NO	Unchanged
18	No	1y	F	Negative	NA	Negative	Negative	Negative	Negative	NA	Nocardiopsis dassonvillei-235(18.85)|Mycoplasma pneumoniae-14(1.12)|Primate bocaparvovirus 1-684(100.00)	Negative	Mycoplasma pneumoniae (Azithromycin treatment was effective) , Human bocavirus	NO	NO	Unchanged
19	No	2y	M	Negative	NA	Positive	>1：1280	Negative	Negative	NA	Haemophilus parainfluenzae-3(0.012)|Streptococcus pneumoniae-12(0.048)|Mycoplasma pneumoniae-24446(97.82)	Mycoplasma pneumoniae-1069(79.13)|Human orthopneumovirus-9960(100.00)	Mycoplasma pneumoniae, Respiratory syncytial virus	NO	NO	Unchanged

20	No	8y	F	Negative	NA	Positive	＞1：1280	Negative	Negative	NA	Mycoplasma pneumoniae-517(85.03)	Mycoplasma pneumoniae-63(32.47)	Mycoplasma pneumoniae	YES	N0	Unchanged
21	No	5y	M	Negative	Mycoplasma pneumoniae	Positive	＞1：1280	Negative	Negative	NA	Mycoplasma pneumoniae-3628(99.29)	Mycoplasma pneumoniae-42184(98.70)	Mycoplasma pneumoniae	NO	NO	Unchanged
22	No	2y	M	Negative	NA	Negative	1：160	Negative	Negative	NA	Mycoplasma pneumoniae-15889(99.84)	Mycoplasma pneumoniae-62951(99.79)	Mycoplasma pneumoniae	NO	NO	Unchanged
23	No	12y	M	Negative	NA	Negative	1:320	Negative	Negative	NA	Mycoplasma pneumoniae-123867(99.79)	Mycoplasma pneumoniae-124108(99.80)	Mycoplasma pneumoniae	NO	A2063G	Unchanged
24	No	4y	F	Negative	Mycoplasma pneumoniae	Negative	Negative	Negative	Negative	NA	Mycoplasma pneumoniae-5501(95.70)	Mycoplasma pneumoniae-38102(97.56)	Mycoplasma pneumoniae	NO	NO	Unchanged
25	No	16y	F	Negative	Mycoplasma pneumoniae	Positive	＞1：1280	Negative	Negative	NA	Streptococcus pneumoniae-168(0.02)|Mycoplasma pneumoniae-29999(4.14) |Streptococcus pseudopneumoniae-	Mycoplasma pneumoniae-7721(40.87)|Human gammaherpesvirus 4-3(17.65)	Mycoplasma pneumoniae	YES	A2063G	Unchanged
26	No	6y	F	Negative	Mycoplasma pneumoniae	Positive	＞1：1280	Negative	Negative	NA	Mycoplasma pneumoniae-374840(99.99)	Mycoplasma pneumoniae-7892(24.02)	Mycoplasma pneumoniae	NO	A2063G	Unchanged

27	No	8y	M	Negative	Mycoplasma pneumoniae	Positive	＞1：1280	Negative	Negative	NA	Streptococcus pneumoniae-8(0.0014)|Mycoplasma pneumoniae-559781(99.93) |Achromobacter xylosoxidans-25(0.0045)	Mycoplasma pneumoniae-67(4.75)	Mycoplasma pneumoniae	NO	A2063G	Unchanged
28	No	10y	M	Negative	Mycoplasma pneumoniae	Positive	＞1：1280	Negative	Negative	NA	Haemophilus influenzae-2467(13.04)|Mycoplasma pneumoniae-15649(82.72)	Haemophilus influenzae-873(60.92)|Mycoplasma pneumoniae-271(18.91)	Mycoplasma pneumoniae, Haemophilus influenzae	YES	NO	Unchanged
29	No	10y	F	Negative	Mycoplasma pneumoniae	Positive	1：1280	Negative	Negative	NA	Mycoplasma pneumoniae-245(71.22) |Streptococcus pneumoniae-7(2.03)|Streptococcus pseudopneumoniae-	Mycoplasma pneumoniae-382(73.18)	Mycoplasma pneumoniae	NO	NO	Unchanged
30	No	5y	F	Negative	Mycoplasma pneumoniae	Positive	＞1：1280	Negative	Negative	NA	Haemophilus influenzae-24(0.63)|Haemophilus parainfluenzae-13(0.34)|Mycoplasma pneumoniae-3518(92.92)	Mycoplasma pneumoniae-40(5.15)	Mycoplasma pneumoniae	YES	NO	Unchanged
31	No	9y	F	Negative	Mycoplasma pneumoniae	Positive	＞1：1280	Negative	Negative	Negative	Mycoplasma pneumoniae-8386(99.70)	Mycoplasma pneumoniae-1089(17.29)	Mycoplasma pneumoniae	YES	NO	Unchanged
32	No	4y	F	Negative	Mycoplasma pneumoniae, Human metap	Negative	Negative	Negative	Negative	NA	Haemophilus parainfluenzae-3(0.03)|Staphylococcus aureus-8(0.09)|Streptococcus pneumoniae-129(1.47)|Mycoplasma	Human metapneumovirus-92(96.84)	Mycoplasma pneumoniae, Human metapneumovirus	NO	NO	Unchanged
33	No	9y	F	Negative	Mycoplasma pneumoniae	Positive	1：1280	Negative	Negative	NA	Enterococcus faecium-3(0.0032)|Mycoplasma pneumoniae-48714(99.94)	Mycoplasma pneumoniae-62568(99.78)	Mycoplasma pneumoniae	YES	A2063G	Added linezolid (mNGS-Enterococcus faecium)（NO）

34	No	10y	F	Negative	Mycoplasma pneumoniae	Positive	1：640	Negative	Negative	NA	Mycoplasma pneumoniae-21815(99.96)	Mycoplasma pneumoniae-55128(99.41)	Mycoplasma pneumoniae	NO	NO	Unchanged
35	No	3y	M	Negative	NA	Positive	1：1280	NA	Negative	NA	Streptococcus pneumoniae-3(0.054)|Mycoplasma pneumoniae-5514(99.15)|Human betaherpesvirus 7-2(66.67)	Mycoplasma pneumoniae-33915(99.29)	Mycoplasma pneumoniae	NO	NO	Unchanged
36	No	1y	F	Negative	NA	Positive	>1：1280	Negative	Negative	NA	Streptococcus pneumoniae-6(0.15)|Mycoplasma pneumoniae-3836(98.31)	Mycoplasma pneumoniae-531(72.74)	Mycoplasma pneumoniae	NO	NO	Unchanged
37	No	5y	M	Negative	NA	Positive	>1：1280	Negative	Negative	NA	Mycoplasma pneumoniae-665(96.38)	Mycoplasma pneumoniae-67(39.18)	Mycoplasma pneumoniae	NO	NO	Unchanged
38	No	5y	F	Negative	NA	Positive	>1：1280	NA	Negative	NA	Mycoplasma pneumoniae-938(97.81)	Mycoplasma pneumoniae-703(91.42)	Mycoplasma pneumoniae	NO	NO	Unchanged
39	No	3y	M	Negative	Mycoplasma pneumoniae	Positive	>1：1280	Negative	Negative	Negative	Haemophilus parainfluenzae-3(0.018)|Streptococcus pneumoniae-4(0.024)	Negative	Mycoplasma pneumoniae	NO	NO	Unchanged
40	No	10y	F	Negative	Mycoplasma pneumoniae	Negative	1:320	Negative	Negative	NA	Mycoplasma pneumoniae-5750(99.98)	Mycoplasma pneumoniae-46287(99.77)	Mycoplasma pneumoniae	NO	NO	Unchanged

41	No	3y	F	Negative	Mycoplasma pneumoniae	Negative	1：160	Negative	Negative	NA	Mycoplasma pneumoniae-259004(99.93)	Mycoplasma pneumoniae-68547(99.86)	Mycoplasma pneumoniae	NO	A2063G	Unchanged
42	No	7m	M	Negative	Mycoplasma pneumoniae	NA	1：160	Negative	Negative	NA	Finegoldia magna-171(0.31)|Staphylococcus aureus-15(0.027)|Streptococcus pneumoniae-9(0.016)|Mycoplasma pneumoniae-	Finegoldia magna-5(0.0084)|Mycoplasma pneumoniae-58989(99.42)	Mycoplasma pneumoniae	NO	A2063G	Unchanged
43	No	4y	M	Negative	Mycoplasma pneumoniae	Positive	1：1280	Negative	Negative	NA	Streptococcus pneumoniae-17(0.029)|Mycoplasma pneumoniae-56959(99.25)|Streptococcus pseudopneumoniae-	Mycoplasma pneumoniae-147241(99.83)	Mycoplasma pneumoniae	NO	A2063G	Added azithromycin(mNGS-Mycoplasma pneumoniae)(YES)
44	No	6y	F	Negative	Mycoplasma pneumoniae	Negative	Negative	Negative	Negative	NA	Streptococcus pneumoniae-1(0.04)|Mycoplasma pneumoniae-7631(99.13)	Mycoplasma pneumoniae-10231(98.62)	Mycoplasma pneumoniae	YES	NO	Unchanged
45	No	9y	F	Negative	NA	Negative	1:640	Negative	Negative	NA	Mycoplasma pneumoniae-9337(99.97)	Mycoplasma pneumoniae-54423(99.77)	Mycoplasma pneumoniae	YES	NO	Unchanged
46	No	9y	M	Negative	Mycoplasma pneumoniae	Negative	Negative	Negative	Negative	NA	Mycoplasma pneumoniae-14517(99.79)	Mycoplasma pneumoniae-783(74.71)	Mycoplasma pneumoniae	NO	NO	Unchanged
47	No	6m	F	Negative	Mycoplasma pneumoniae	Positive	1:640	Negative	Negative	NA	Mycoplasma pneumoniae-340(95.77)	Mycoplasma pneumoniae-297(11.61)	Mycoplasma pneumoniae	NO	NO	Unchanged

48	No	9m	M	Moraxella catarrhalis	Mycoplasma pneumoniae	Negative	Negative	Negative	Negative	NA	Streptococcus pneumoniae-43(1.38)|Mycoplasma pneumoniae-3009(96.47)|Streptococcus pseudopneumoniae-	Mycoplasma pneumoniae-13(3.42)	Mycoplasma pneumoniae, Moraxella catarrhalis	NO	NO	Unchanged
49	No	5y	F	Negative	Mycoplasma pneumoniae	Negative	Negative	Negative	Negative	NA	Mycoplasma pneumoniae-54(71.05)	Negative	Mycoplasma pneumoniae	NO	NO	Added azithromycin(mNGS-Mycoplasma pneumoniae)(YES)
50	No	8y	F	Negative	Negative	Positive	1：640	Negative	Negative	NA	Streptococcus pneumoniae-5(0.04)|Mycoplasma pneumoniae-12186(96.26)	Negative	Mycoplasma pneumoniae	YES	NO	Unchanged
51	No	8y	F	Negative	NA	Positive	1:1280	Negative	Negative	NA	Mycoplasma pneumoniae-726(96.67)	Mycoplasma pneumoniae-33(9.94)	Mycoplasma pneumoniae	NO	NO	Unchanged
52	No	1y	M	Negative	NA	Positive	1：1280	Negative	Negative	Negative	Haemophilus influenzae-77(0.59),|Human bocavirus type I-675(99.85%)	Haemophilus influenzae-61(12.45)|Primate bocaparvovirus 1-8(88.89)	Mycoplasma pneumoniae, Haemophilus influenzae(DNA and RNA results were consistent), Human bocavirus	NO	NO	Added azithromycin(MP IgG antibody titer-Mycoplasma pneumoniae)(YES)
53	No	5y	F	Negative	NA	Negative	Negative	Negative	Negative	NA	Haemophilus parainfluenzae-6(0.095)|Staphylococcus aureus-38(0.60)|Streptococcus pneumoniae-90.14|Mycoplasma	Mycoplasma pneumoniae-26487(99.52)	Mycoplasma pneumoniae	NO	NO	Added azithromycin(mNGS-Mycoplasma pneumoniae)(YES)
54	No	7y	M	Negative	Mycoplasma pneumoniae	Positive	＞1：1280	Negative	Negative	NA	Mycoplasma pneumoniae-127751	Mycoplasma pneumoniae-114017(99.57)	Mycoplasma pneumoniae	YES	A2063G	Unchanged

55	No	4y	F	Negative	Mycoplasma pneumoniae	Negative	Negative	Negative	Negative	NA	Haemophilus influenzae-31(53.45)	Negative	Mycoplasma pneumoniae,Haemophilus influenzae	NO	NO	Unchanged
56	No	5y	M	Negative	Human parainfluenza virus	Positive	1：1280	Negative	Negative	NA	Haemophilus parainfluenzae-77(8.50)|Streptococcus pneumoniae-8(0.88)	Haemophilus parainfluenzae-4(1.83)|Human respirovirus 3-47922(99.99)	Mycoplasma pneumoniae,Haemophilus parainfluenzae, Human parainfluenza virus	NO	NO	Unchanged
57	No	3y	F	Negative	Human parainfluenza virus, Respiratory	Positive	＞1：1280	Negative	Negative	NA	Haemophilus parainfluenzae-266(2.60)|Staphylococcus aureus-13(0.13)|Streptococcus pneumoniae-812(7.93)|Streptococcus	Haemophilus parainfluenzae-42(6.91)|Human respirovirus 3-3258(48.01)|Human orthopneumovirus-3526(51.96)	Mycoplasma pneumoniae,Haemophilus parainfluenzae, Human parainfluenza virus, Respiratory syncytial virus	NO	NO	Unchanged
58	No	5y	M	Negative	Human parainfluenza virus	Positive	Negative	Negative	Negative	NA	Haemophilus parainfluenzae-23(5.48)	Human respirovirus 3-2957(99.93)	Human parainfluenza virus, Haemophilus parainfluenzae	NO		Changed ceftriaxone to cefepime(mNGS-Haemophilus parainfluenzae)(YES)
59	No	3y	M	Negative	NA	Negative	NA	NA	Negative	NA	Pseudomonas aeruginosa-1372(0.058)|Moraxella catarrhalis-10(0.0004)|Haemophilus parainfluenzae-205819(8.68)|Staphylococcus aureus-	Haemophilus parainfluenzae-563(7.95)|Parechovirus A-48(92.30)	Haemophilus parainfluenzae(DNA and RNA results were consistent), Streptococcus pneumoniae(Changing the antibiotic	NO		Changed to ceftriaxone (mNGS--Haemophilus parainfluenzae & Streptococcus pneumoniae)(YES)
60	No	10y	F	Negative	NA	Negative	1：160	Negative	Negative	Positive	Haemophilus parainfluenzae-107(0.45)	Haemophilus parainfluenzae-318(0.52)|Human orthopneumovirus-90(17.11)	Haemophilus parainfluenzae(DNA and RNA results were consistent), Respiratory syncytial virus	NO		Added azithromycin(MP IgG antibody titer-Mycoplasma pneumoniae)(NO)
61	No	2y	M	Negative	Negative	Negative	Negative	Negative	Negative	NA	Bordetella parapertussis-48(30.38)|Haemophilus parainfluenzae-7(4.43)|Human betaherpesvirus 5-10(45.45)	Haemophilus parainfluenzae-3(2.44)|Human betaherpesvirus 5-12(63.16)	Haemophilus parainfluenzae，Bordetella parapertussis	NO		Unchanged

62	Pulmonary hemosiderosis	16y	M	Negative	NA	Negative	Negative	Negative	Negative	NA	Haemophilus parainfluenzae-504(1.68) |Staphylococcus aureus-159(0.53)|Streptococcus pneumoniae-5(0.017)	Negative	Haemophilus parainfluenzae	NO		Unchanged
63	No	3m	F	Negative	Respiratory syncytial virus	Negative	Negative	Negative	Negative	NA	Haemophilus parainfluenzae-19828(8.44) |Mycoplasma pneumoniae-13(0.0055)|Ralstonia mannitolilytica-	Haemophilus parainfluenzae-357(43.54)|Ralstonia mannitolilytica-238(29.02)|Human orthopneumovirus-41(18.64)	Haemophilus parainfluenzae, Respiratory syncytial virus	NO		Unchanged
64	Primary ciliary dyskinesia syndrome	8y	F	Negative	NA	Negative	Negative	Negative	Negative	NA	Haemophilus parainfluenzae-52(8.55)	Haemophilus parainfluenzae-29(5.31)	Haemophilus parainfluenzae	NO		Unchanged
65	Aspiration pneumonia, chromosomal	9m	M	Pseudomonas aeruginosa	NA	Negative	Negative	Negative	Negative	Positive	Pseudomonas aeruginosa-355087(50.19)|Klebsiella pneumoniae-4(0.0006)|Haemophilus parainfluenzae-12235(1.73) |Acinetobacter	Pseudomonas aeruginosa-1236(42.95)|Haemophilus parainfluenzae-197(6.85)	Pseudomonas aeruginosa, Haemophilus parainfluenzae	NO		Unchanged
66	No	3y	F	Negative	NA	Positive	1：160	Negative	Negative	NA	Moraxella catarrhalis-1061(0.45)|Haemophilus parainfluenzae-9735(4.16)| Streptococcus pneumoniae-1863(0.80)|Enterococcus	Moraxella catarrhalis-28(0.57)|Haemophilus parainfluenzae-212(4.35)|Human gammaherpesvirus 4-110(55.28)| RhinovirusB-	Moraxella catarrhalis, Haemophilus parainfluenzae (DNA and RNA results were consistent), Rhinovirus	NO		Unchanged
67	No	5m	M	Negative	Negative	Negative	Negative	Negative	Negative	NA	Staphylococcus aureus-32(0.012)|Human betaherpesvirus 5-8(100.00)	Human betaherpesvirus 5-618(100.00)	Staphylococcus aureus（Changing the antibiotic worked）, Cytomegalovirus humanbeta5	NO		Added cefazolin and ganciclovir(mNGS-Staphylococcus aureus & Cytomegalovirus humanbeta5)(YES)
68	No	3m	F	Negative	Rhinovirus	Negative	Negative	Negative	Negative	NA	Streptococcus pneumoniae-42(0.009)	RhinovirusA-35(100.00)	Rhinovirus, Streptococcus pneumoniae (Changing the antibiotic worked)	NO		Added amoxicillin clavulanate potassium(mNGS-Streptococcus pneumoniae)(YES)

69	No	4y	M	Negative	Negative	Negative	Negative	Negative	Negative	NA	Streptococcus pneumoniae-14(0.50)|Achromobacter xylosoxidans-23(0.83)|Corynebacterium resistens-4(0.14)|[Candida]	Negative	Streptococcus pneumoniae (Clinically appropriate and therapeutically effective)	NO		Unchanged
70	No	6y	M	Negative	Negative	Positive	1:320	Negative	Negative	NA	Pseudomonas aeruginosa-241(1.42)|Staphylococcus aureus-623(3.67)|Streptococcus pneumoniae-6391(37.64)	Negative	Streptococcus pneumoniae	NO		Added amoxicillin clavulanate potassium(mNGS-Streptococcus pneumoniae)(YES)
71	Pulmonary hemosiderosis	1y	M	Negative	Respiratory syncytial virus	Negative	Negative	Negative	Negative	NA	Human mastadenovirus C-31(100.00)	Human orthopneumovirus-292(98.98)	Respiratory syncytial virus, Adenovirus	NO		Unchanged
72	No	6y	F	Negative	Human parainfluenza virus	Negative	Negative	Negative	Negative	NA	Streptococcus pneumoniae-4(9.30)	Human respirovirus 3-7(43.75)	Streptococcus pneumoniae(Clinically appropriate and therapeutically effective), Human parainfluenza virus	NO		Changed to penicillin and amoxicillin clavulanate potassium(mNGS-Streptococcus pneumoniae)(YES)
73	No	1y	M	Negative	Human metapneumovirus	Negative	Negative	Negative	Negative	NA	Haemophilus parainfluenzae-59(3.64)|Stenotrophomonas maltophilia-23(1.42)|Mycobacterium xenopi-4(0.25)	Human metapneumovirus-27206(100.00)	Human metapneumovirus	NO		Unchanged
74	No	6y	F	Negative	Rhinovirus	Negative	Negative	Negative	Negative	NA	Negative	RhinovirusC-172(98.85)	Rhinovirus	NO		Unchanged
75	Cystic fibrosis	14y	M	Pseudomonas aeruginosa	Negative	Negative	1：160	Negative	Negative	Negative	Pseudomonas aeruginosa-450311(99.99)|Penicillium digitatum-3(23.08)	Negative	Pseudomonas aeruginosa, Penicillium digitatum	NO		Unchanged

76	No	6y	M	Negative	Negative	Negative	Negative	Negative	Negative	NA	Candida tropicalis-5(7.58)	Negative	Negative	NO		Unchanged
77	No	2y	F	Negative	Negative	Negative	Negative	Negative	Negative	NA	Haemophilus influenzae-11(100.00)	Haemophilus influenzae-32(24.24)	Haemophilus influenzae	NO		Changed to cefoperazone/ sulbactam sodium(mNGS-Haemophilus influenzae)(YES)
78	Pulmonary hemosiderosis	4y	F	Negative	Human metapneumovirus	Negative	Negative	Negative	Negative	NA	Human betaherpesvirus 5-3(100.00)	Human metapneumovirus-6986(99.94)	Human metapneumovirus	NO		Unchanged
79	No	1y	M	Negative	Respiratory syncytial virus	Negative	Negative	Negative	Negative	NA	Negative	Human orthopneumovirus-27892(100.00)	Respiratory syncytial virus	NO		Unchanged
80	Tracheomalacia (with severe stenosis)	9m	F	Negative	Negative	Negative	Negative	Negative	Negative	NA	Streptococcus pneumoniae-3(0.17)|Tropheryma whipplei-1670(95.32)	Streptococcus pneumoniae-3(0.08)|Tropheryma whipplei-5(0.13)	Tropheryma whipplei, Streptococcus pneumoniae (DNA and RNA results were consistent & Changing the antibiotic worked)	NO		Added TMP-SMZ(mNGS-Tropheryma whipplei)(YES)
81	No	4y	M	Negative	Negative	Negative	Negative	Negative	Negative	NA	Haemophilus influenzae-84(69.42)|Human betaherpesvirus 5-272(98.55)	Human betaherpesvirus 5-5(83.33)	Haemophilus influenzae	NO		Unchanged
82	No	1y	F	Negative	Rhinovirus	Negative	Negative	Negative	Negative	NA	Haemophilus influenzae-152(71.03)|Human betaherpesvirus 5-15(68.18)	Haemophilus influenzae-27(7.22)|Rhinovirus C-3471(97.31)	Haemophilus influenzae, Rhinovirus	NO		Added azithromycin and ceftriaxone(mNGS-Haemophilus influenzae & Rhinovirus)(YES)

83	Tracheomalacia (after tracheoesophageal	2y	M	Negative	Human metapneumovirus	Negative	Negative	Negative	Negative	NA	Tropheryma whipplei-11(44.00)	Human metapneumovirus-74(98.67)	Tropheryma whipplei, Human metapneumovirus	NO		Added TMP-SMZ(mNGS-Tropheryma whipplei)(YES)
84	No	5Y	M	Negative	Negative	Negative	Negative	Negative	Negative	NA	Klebsiella pneumoniae-18(5.22)|Mycoplasma pneumoniae-8(2.32)	Negative	Negative	NO		Unchanged
85	Growth retardation, aspiration pneum	9m	F	Escherichia coli	Negative	Negative	Negative	Negative	Negative	NA	Escherichia coli-1336(16.54)|Streptococcus pneumoniae-322(3.99)|Human betaherpesvirus 5-65(100.00)	Negative	Escherichia coli, Streptococcus pneumoniae(Changing the antibiotic worked)	NO		Changed to cefoperazone/sulbactam sodium(mNGS-Escherichia coli)(YES)
86	No	1y	M	Negative	Negative	Negative	Negative	Negative	Negative	Negative	Haemophilus influenzae-1652(36.79) |Enterococcus faecium-6(0.34)|Candida parapsilosis-5(2.99)	Haemophilus influenzae-53(19.34)|Pegivirus C-21(84.00)	Haemophilus influenzae	NO		Added linezolid (mNGS-Enterococcus faecium)(NO)
87	Bronchopulmonary dysplasia	6y	M	Negative	Rhinovirus	Negative	Negative	Negative	Negative	NA	Staphylococcus aureus-5(4.42)	Negative	Staphylococcus aureus(Clinically appropriate and therapeutically effective), Rhinovirus	NO		Unchanged
88	No	7y	F	Negative	Human parainfluenza virus	Negative	Negative	Negative	Negative	NA	Haemophilus influenzae-194(87.39)	Haemophilus influenzae-8(0.44)|Human respirovirus 3-571(100.00)	Haemophilus influenzae, Human parainfluenza virus	NO		Unchanged
89	No	1y	M	Negative	Negative	Negative	Negative	Negative	Negative	NA	Haemophilus influenzae-3134(10.32)| Streptococcus pneumoniae-1738(5.72)|Mycoplasma pneumoniae-3(0.0099)|Streptococcus	Haemophilus influenzae-1587(69.42)|Human betaherpesvirus 5-5(1.39)|Human gammaherpesvirus 4-354(98.61)	Haemophilus influenzae	NO		Unchanged

90	No	2y	M	Negative	Respiratory syncytial virus	Negative	Negative	Negative	Negative	NA	Haemophilus influenzae-7(14.29)	Human orthopneumovirus-5040(100.00)	Haemophilus influenzae, Respiratory syncytial virus	NO		Unchanged
91	Growth retardation	5y	M	Methicillin-resistant Staphylococcus aureus	NA	Negative	NA	NA	Negative	NA	Mycoplasma pneumoniae-6(6.52)|Staphylococcus aureus-5(5.43)	Human respirovirus 1-885(99.89)	Methicillin-resistant Staphylococcus aureus (Consistent with culture results), Human parainfluenza virus	NO		Changed to vancomycin (Culture-Methicillin-resistant Staphylococcus aureus)(YES)
92	No	15y	F	Enterobacter cloacae	NA	Negative	Negative	NA	Negative	Negative	Finegoldia magna-3(0.027)|Streptococcus pneumoniae-16(0.14)|Mycoplasma pneumoniae-6(0.053)|Rhizopus microsporus-	Rhizopus microsporus-82(38.32)|Human coronavirus 229E-24(92.31)	Rhizopus microsporus, Enterobacter cloacae, Coronavirus	NO		Added voriconazole(mNGS-Rhizopus microsporus), added levofloxacin (Culture-Enterobacter cloacae) (YES)
93	No	3y	F	Negative	NA	Negative	NA	NA	Negative	Negative	Streptococcus pneumoniae-30(0.21)	RhinovirusA-40(100.00)	Streptococcus pneumoniae (Changing the antibiotic worked), Rhinovirus	NO		Changed to amoxicillin clavulanate potassium(mNGS-Streptococcus pneumoniae)(YES)
94	No	2y	M	Negative	NA	Positive	NA	NA	Negative	NA	Negative	Human respirovirus 1-80(96.39)	Human parainfluenza virus	NO		Unchanged
95	Cystic fibrosis	16y	F	Pseudomonas aeruginosa	NA	Negative	NA	NA	Negative	Negative	Pseudomonas aeruginosa-260242(99.92)|Aspergillus fumigatus-32(35.96)	Pseudomonas aeruginosa-26105(95.57)	Pseudomonas aeruginosa, Aspergillus	NO		Added voriconazole, TMP-SMZ and tobramycin nebulisation(mNGS-Pseudomonas aeruginosa & Aspergillus)(YES)
96	No	11m	M	Negative	NA	Negative	NA	Negative	Negative	NA	Haemophilus parahaemolyticus-10(0.21)|Streptococcus pneumoniae-60(1.25)|Streptococcus pseudopneumoniae-13(0.27)|Human	Human bocavirus type I-1104(100.00)	Streptococcus pneumoniae(Clinically appropriate and therapeutically effective), Human bocavirus	NO		Unchanged

<table>
<tr><td>97</td><td>Cystic fibrosis</td><td>17y</td><td>F</td><td>Pseudomonas aeruginosa</td><td>NA</td><td>NA</td><td>NA</td><td>NA</td><td>Negative</td><td>Negative</td><td>Pseudomonas aeruginosa-185284(99.96)|Aspergillus flavus-26(10.20)|Aspergillus fumigatus-89(34.90)</td><td>Pseudomonas aeruginosa-27384(91.17)</td><td>Pseudomonas aeruginosa，Aspergillus flavus，Aspergillus fumigatus</td><td>NO</td><td></td><td>Added voriconazole(mNGS-Aspergillus)(YES)</td></tr>
<tr><td>98</td><td>No</td><td>3y</td><td>F</td><td>Negative</td><td>NA</td><td>Negative</td><td>NA</td><td>Negative</td><td>Negative</td><td>NA</td><td>Haemophilus influenzae-114(6.17)</td><td>Haemophilus influenzae-122(18.15)</td><td>Haemophilus influenzae (DNA and RNA results were consistent)</td><td>NO</td><td></td><td>Unchanged</td></tr>
<tr><td>99</td><td>No</td><td>7y</td><td>F</td><td>Negative</td><td>NA</td><td>Negative</td><td>NA</td><td>Negative</td><td>Negative</td><td>NA</td><td>Haemophilus influenzae-42(82.35)</td><td>Haemophilus influenzae-81(33.33)|Rhinovirus A-141(76.63)</td><td>Haemophilus influenzae, Rhinovirus</td><td>NO</td><td></td><td>Unchanged</td></tr>
<tr><td>100</td><td>No</td><td>2y</td><td>M</td><td>Negative</td><td>NA</td><td>Negative</td><td>Negative</td><td>Negative</td><td>Negative</td><td>NA</td><td>Haemophilus parahaemolyticus-28(0.10)|Streptococcus pneumoniae-88(0.33)|Tropheryma whipplei-797(2.95)|Leuconostoc</td><td>Negative</td><td>Human bocavirus, Streptococcus pneumoniae(Clinically appropriate and therapeutically effective)</td><td>NO</td><td></td><td>Unchanged</td></tr>
<tr><td>101</td><td>No</td><td>14y</td><td>M</td><td>Staphylococcus aureus</td><td>NA</td><td>Negative</td><td>Negative</td><td>Negative</td><td>Negative</td><td>Negative</td><td>Staphylococcus aureus-27255(99.96)</td><td>Staphylococcus aureus-12908(98.89)</td><td>Staphylococcus aureus</td><td>NO</td><td></td><td>Unchanged</td></tr>
<tr><td>102</td><td>No</td><td>9m</td><td>M</td><td>Streptococcus pneumoniae</td><td>NA</td><td>Negative</td><td>Negative</td><td>Negative</td><td>Negative</td><td>NA</td><td>Streptococcus pneumoniae-7(0.67)|Human betaherpesvirus 6-3(42.86)</td><td>Negative</td><td>Streptococcus pneumoniae(Consistent with culture results)</td><td>NO</td><td></td><td>Unchanged</td></tr>
<tr><td>103</td><td>Aspiration pneumonia</td><td>2m</td><td>F</td><td>Negative</td><td>Chlamydia, Human parainfluenza virus</td><td>Negative</td><td>Negative</td><td>NA</td><td>Negative</td><td>NA</td><td>Chlamydia trachomatis-2041(96.46)|Human betaherpesvirus 5-19(82.61)</td><td>Chlamydia trachomatis-1418(89.29)|Human betaherpesvirus 5-37(26.24)|Human respirovirus 3-103(73.05)</td><td>Chlamydia trachomatis, Cytomegalovirus humanbeta5, Human parainfluenza virus</td><td>NO</td><td></td><td>Unchanged</td></tr>
</table>

104	Aspiration pneumonia, chromosomal	8m	M	Multidrug-resistant Streptococcus pneumo	NA	Negative	Negative	Negative	Negative	Positive	Streptococcus pneumoniae-6975(8.16)|Corynebacterium striatum-20(0.023)	Negative	Multidrug-resistant Streptococcus pneumoniae, Aspergillus（血G试验和GM试验均为阳性，临床抗真菌治疗有效）	NO		Added vancomycin (Culture-Multidrug-resistant Streptococcus pneumoniae)(YES)
105	Primary ciliary dyskinesia syndrome	14y	F	Negative	NA	Negative	Negative	Negative	Negative	Negative	Haemophilus influenzae-18673(99.11)	Haemophilus influenzae-24906(98.87)	Haemophilus influenzae	NO		Unchanged
106	No	6m	M	Negative	Respiratory syncytial virus	Negative	Negative	Negative	Negative	NA	Streptococcus pneumoniae-2945(0.91)|Candida albicans-2188(39.72)|Human betaherpesvirus 5-5607(99.98)	Human betaherpesvirus 5-47(0.026)|Human orthopneumovirus-179430(99.97)	Streptococcus pneumoniae, Respiratory syncytial virus	NO		Unchanged
107	Cystic fibrosis	1y	F	Methicillin-resistant Staphylococcus aureus	NA	Negative	Negative	Negative	Negative	NA	Staphylococcus aureus-73325(99.94)|Aspergillus terreus-67(54.92)	Staphylococcus aureus-4260(94.27)	Methicillin-resistant Staphylococcus aureus, Aspergillus	NO		Changed to linezolid (mNGS & culture-Methicillin-resistant Staphylococcus aureus)(YES)
108	No	7y	F	Negative	Negative	Negative	Negative	Negative	Negative	Negative	Negative	Negative	Negative	NO		Unchanged
109	No	9y	M	Negative	NA	Negative	Negative	Negative	Negative	NA	Negative	Negative	Negative	NO		Unchanged
110	No	6y	M	Negative	NA	Negative	Negative	Negative	Negative	Positive	Streptococcus pneumoniae-7(2.14)	Human orthopneumovirus-6(13.04)	Streptococcus pneumoniae, Respiratory syncytial virus	NO		Unchanged

111	No	1y	F	Negative	NA	Negative	Negative	Negative	Negative	NA	Haemophilus parainfluenzae-181(0.40)|Streptococcus pneumoniae-1810(3.99)|Enterococcus faecalis-106(0.23)|Mycolicibacter	Negative	Streptococcus pneumoniae(Clinically appropriate and therapeutically effective)	NO		Unchanged
112	No	4y	M	Negative	Human metapneumovirus	Negative	Negative	Negative	Negative	NA	Tropheryma whipplei-199(43.93)	Tropheryma whipplei-54(13.20)|Human metapneumovirus-436(96.46)	Human metapneumovirus	NO		Unchanged
113	Tracheomalacia (after tracheoesophageal	2y	F	Negative	NA	Negative	Negative	Negative	Negative	NA	Escherichia coli-194(0.033)|Klebsiella pneumoniae-5(0.0009)|Streptococcus pneumoniae-42505(7.25)|Streptococcus	Candida albicans-3(10.34)|Human gammaherpesvirus 4-67(100.00)	Streptococcus pneumoniae, Streptococcus pseudopneumoniae, Candida albicans	NO		Added fluconazol(mNGS-Candida albicans)(YES)
114	No	2y	F	Negative	Respiratory syncytial virus	Negative	Negative	Negative	Negative	NA	Moraxella catarrhalis-3(1.06)|Staphylococcus aureus-29(10.21)|Mycoplasma pneumoniae-6(2.11)|Nocardia cyriacigeorgica-4(1.41)	Human orthopneumovirus-4856(99.98)	Respiratory syncytial virus	NO		Unchanged
115	No	1y	F	Negative	Human parainfluenza virus	Negative	Negative	NA	Negative	NA	Klebsiella aerogenes-3(0.0031)|Streptococcus pneumoniae-1886(1.97)|Mycoplasma pneumoniae-3(0.0031)|Streptococcus pseudopneumoniae-	Human respirovirus 3-2283(99.91)	Streptococcus pneumoniae, Streptococcus pseudopneumoniae, Human parainfluenza virus	NO		Unchanged
116	No	1y	M	Negative	Negative	Negative	Negative	Negative	Negative	NA	Streptococcus pneumoniae-9(3.61)|Streptococcus pseudopneumoniae-4(1.61)|Pneumocystis jiroveci-4(30.77)	Pneumocystis jiroveci-686(96.21)	Pneumocystis jiroveci	NO		Added TMP-SMZ(mNGS-Pneumocystis jiroveci)(YES)
117	No	2y	F	Haemophilus influenzae	Human parainfluenza virus	Positive	Negative	Negative	Negative	NA	Moraxella catarrhalis-1565(98.49)	Human respirovirus 3-1615(100.00)	Moraxella catarrhalis, Haemophilus influenzae, Human parainfluenza virus	NO		Unchanged

118	No	3y	F	Negative	Negative	Negative	Negative	Negative	Negative	NA	Negative	Negative	Negative	NO		Unchanged
119	Primary ciliary dyskinesia syndrome	12y	M	Negative	Rhinovirus	NA	Negative	Negative	Negative	NA	Haemophilus influenzae-68(78.16)	Haemophilus influenzae-61(6.57)|Rhinovirus A-6(60.00)	Haemophilus influenzae, Rhinovirus	NO		Unchanged
120	No	8y	F	Negative	Human parainfluenza virus	Positive	1：160	Negative	Negative	NA	Mycoplasma pneumoniae-4(4.76)	Betacoronavirus 1-37783(99.99)	Coronavirus	NO		Unchanged
121	No	3y	M	Negative	Negative	Negative	Negative	Negative	Negative	NA	Streptococcus pneumoniae-24(58.54)	Negative	Streptococcus pneumoniae	NO		Unchanged
122	No	16y	M	Negative	Negative	Negative	Negative	Negative	Negative	NA	Negative	Negative	Negative	NO		Unchanged
123	No	12m	M	Streptococcus pneumoniae	Rhinovirus	Negative	Negative	Negative	Negative	NA	Streptococcus pneumoniae-470(96.71)|Mycoplasma pneumoniae-6(1.23)	Streptococcus pneumoniae-4555(86.15)|RhinovirusA-253(98.44)	Streptococcus pneumoniae, Rhinovirus	NO		Unchanged
124	No	4m	M	Negative	Negative	Negative	Negative	NA	Negative	NA	Streptococcus pneumoniae-1832(6.09) |Streptococcus pseudopneumoniae-604(2.01)|Human betaherpesvirus 5-	Human betaherpesvirus 5-155(98.73)	Streptococcus pneumoniae, Cytomegalovirus humanbeta5	NO		Added ganciclovir(mNGS-Cytomegalovirus humanbeta5)(YES)

125	No	15y	M	Negative	Human parainfluenza virus	Negative	Negative	NA	Positive	NA	Mycobacterium tuberculosis-12032(99.41)	Mycobacterium tuberculosis-1(0.65)|Human rubulavirus 2-2151(100.00)	Mycobacterium tuberculosis, Human parainfluenza virus	NO		Unchanged, referred to a sentinel hospital for further tuberculosis treatment
126	No	4y	M	Negative	Negative	Negative	Negative	Negative	Negative	NA	Haemophilus influenzae-4(3.36)|Mycoplasma pneumoniae-14(11.76)	Negative	Haemophilus influenzae	NO		Changed to penicillin and amoxicillin clavulanate potassium(mNGS-Haemophilus influenzae)(YES)
127	No	5y	F	Negative	Negative	Positive	1：160	Negative	Negative	NA	Streptococcus pneumoniae-3593(93.89)	Streptococcus pneumoniae-115(30.67)	Streptococcus pneumoniae	NO		Changed to penicillin and amoxicillin clavulanate potassium(mNGS-Streptococcus pneumoniae)(YES)

NOTE: mPCR: multiplex PCR,MP:Mycoplasma pneumonia; T-spot: tuberculosis infection T cell spot; RMPP: refractory M. pneumoniae pneumonia; mNGS: metagenomic next-generation sequencing; NA: not available.
